# Gallic Acid Triggers Iron-Dependent Cell Death with Apoptotic, Ferroptotic, and Necroptotic Features

**DOI:** 10.3390/toxins11090492

**Published:** 2019-08-26

**Authors:** Ho Man Tang, Peter Chi Keung Cheung

**Affiliations:** School of Life Sciences, The Chinese University of Hong Kong, Shatin, New Territories, Hong Kong, China

**Keywords:** apoptosis, cell death, deferoxamine, ferroptosis, gallic acid, necroptosis, necrosulfonamide

## Abstract

Gallic acid (GA) is a natural anti-cancer compound that can be found in many food sources, including edible mushrooms, fruits, and vegetables. Studies generally attribute the anti-cancer activity of GA to the induction of apoptosis. Here, we reported that GA activated iron-dependent cell death mechanisms with apoptotic, ferroptotic, and necroptotic features. Our time-lapse live-cell microscopy study demonstrated that GA could induce coexistence of multiple types of cell death pathways, including apoptosis characterized by mitochondrial cytochrome *c* release and caspase-3 activation, ferroptosis characterized by lipid peroxidation, and necroptosis characterized by the loss of plasma membrane integrity. This GA-induced cell death could be completely suppressed by exposure to an iron chelator deferoxamine, indicating that it is an iron-dependent cell death process. Importantly, MLKL (mixed lineage kinase domain-like protein) inhibitor necrosulfonamide exerted a synergistic effect by increasing the sensitivity of cancer cells to GA. Taken together, our results provide new mechanistic insights, and also suggest new strategies to enhance the efficacy of this natural anti-cancer compound by identifying the agents that can promote or suppress the GA-induced cell death process.

## 1. Introduction

Despite dramatic scientific gains and technological advancement, cancer remains a major and growing public health problem in the world. Anti-cancer treatments, such as chemotherapy and radiotherapy, kill cancer cells by inducing different types of programmed cell death, including apoptosis, which is a natural suicidal process in cells [1,2,3]. Although many primary cancers, including highly fatal metastatic small cell lung carcinoma and melanoma, often response to aggressive cancer therapy [4,5], there are severe side effects, such as cardiotoxicity and blood disorders [6,7,8]. Such side effects exceed the tolerance of the patients, and thus most chemotherapy and radiotherapy are delivered episodically to let patients recover from the side effects between successive treatments. However, cancer cells can repopulate during the interval between cycles of therapy, leading to treatment failure due to cancer recurrence [9,10]. Therefore, there is a pressing need to devise new and effective strategies for fighting cancer, with less side effect on the patients.

Recent studies have shown that some food products contain natural bioactive substances, which have anti-cancer effects, with the potential of minimal side effects. Of particular interest are mushrooms, which have a very long history of being edible and medicinal resources [11,12,13,14], and are now being recognized for their pharmacological potential as a new generation of anti-cancer agents to treat human cancers, including breast, liver, and colon cancer, leukemia, and lymphoma [11,15,16]. Evidence from animal models reveals that extracts from edible mushrooms, with high efficacy, trigger the demise of cancer cells through apoptosis, and suppress their development and progression [11]. Furthermore, this activity occurs with low toxicity to non-cancerous cells in vitro and in vivo. Thus far, such properties have been most thoroughly studied for the high molecular weight (HMW) extracts of non-digestible polysaccharides and polysaccharide-protein complexes [11,12,13,14].

Less well-known is the identity of low molecular weight (LMW) bioactive substances from edible mushrooms and the molecular mechanisms responsible for their anti-cancer bioactivity. Previous studies by our group and others have successfully identified several LMW phenolic compounds, including gallic acid (GA), in the extract of edible mushrooms, that have potential anti-cancer effects [17,18,19]. GA is found in many dietary substances, including edible mushrooms, fruits, herbs, and vegetables [20]. It is generally believed that GA triggers cancer cells to die through apoptosis, as the GA-induced cells display hallmarks of apoptosis, including mitochondrial fragmentation, the release of cytochrome *c* from mitochondria to cytosol, nuclear condensation, DNA damage, and caspase-3 activation [20]. However, the induction of GA to other forms of programmed cell death has not been fully tested.

To harness the discovery of anti-cancer property of GA to develop a new therapeutic intervention, it is important to understand how GA triggers cell death and to identify agents to modulate the lethality of GA. Therefore, we investigated which forms of cell death can be triggered by GA, and screened for the agents that can control the cell death-inducing activity of GA. Our time-lapse live-cell microscopy demonstrated that GA could trigger a novel iron-dependent cell death with apoptotic, ferroptotic, and necroptotic features. Our drug screening also revealed that the cell death-inducing activity of GA could be suppressed by an iron chelator deferoxamine (DFO), and be enhanced by MLKL (mixed lineage kinase domain-like protein) inhibitor necrosulfonamide (NSA). The identification of such compounds could provide mechanistic insights, and suggest new strategies for treating cancers by using this natural anti-cancer bioactive substance isolated from food sources.

## 2. Results

### 2.1. Activation of Apoptotic Cell Death by Gallic Acid

**Figure 1:**
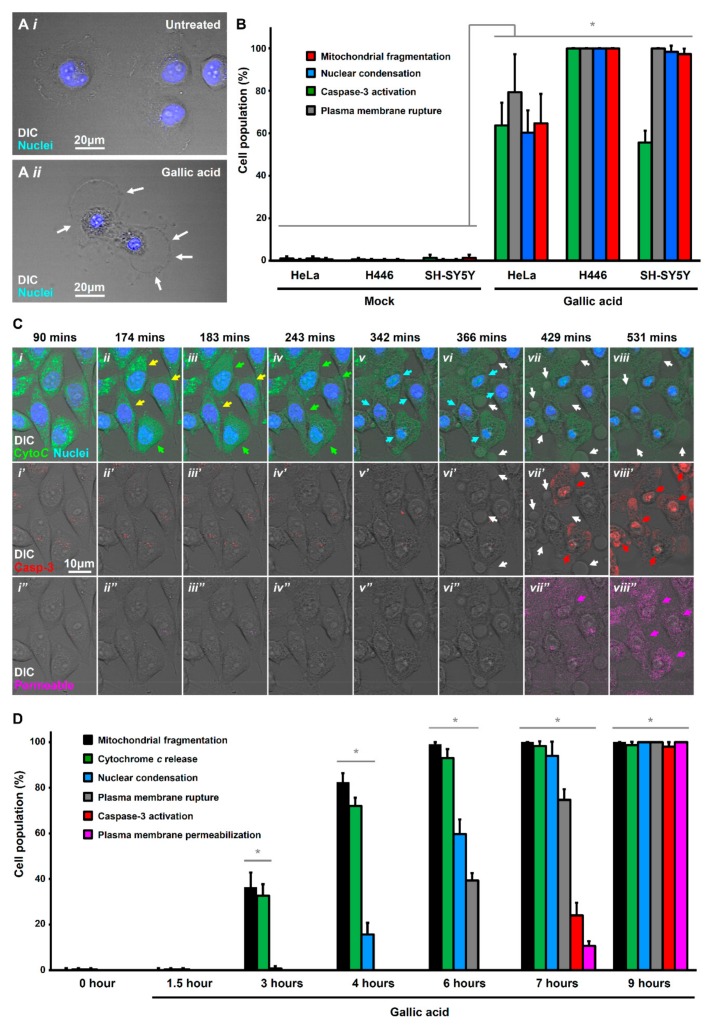
Gallic acid-induced cells display hallmarks of apoptosis and necroptosis. (**A**) Morphology of HeLa cells before (*i*) and after (*ii*) treatment with gallic acid (GA, 50 µg/mL) for 12 h. Cells were stained with Hoechst 33342 to visualize the nucleus. Cell morphology was observed by DIC microscopy. White arrows indicate the plasma membrane rupture. (**B**) Quantification of the cell death events in response to gallic acid induction for 12 h. The percentage in cell population of HeLa, H446, and SH-SY5Y displayed cell death events of mitochondrial fragmentation (red), nuclear condensation (blue), caspase-3 activation (green), plasma membrane rupture (grey) with or without treatment with 50 µg/mL of gallic acid for 12 h. (Mean ± s.d.; *n* = 3). (**C**) Time-lapse live-cell confocal microscopy on cytochrome *c*-GFP expressing HeLa cells treated with 50 µg/mL of gallic acid. Merged images of differential interference contrast (DIC) microscopy, cytochrome *c*-GFP, and nucleus (top row); DIC and caspase substrate (middle row); DIC and plasma membrane-permeable dye (bottom row). Arrows indicate the cells that display mitochondrial fragmentation (yellow), cytochrome *c* release (green), nuclear condensation (blue), caspase-3 activation (red), plasma membrane rupture (white), and permeabilization (pink). Cells were stained with blue nuclear dye Hoechst 33342, before treatment with gallic acid together with NucView 530 Caspase-3substrate (red) and plasma membrane-permeable dye IncuCyte Cytotox red reagent (pink). Supplementary Materials Video S1 is available as the Supplementary Materials. (**D**) Quantification of the cell death events in HeLa cells in response to time-course gallic acid induction. The percentage in population of HeLa cells displayed cell death events of mitochondrial fragmentation (black), cytochrome *c* release (green), nuclear condensation (blue), plasma membrane rupture (grey), caspase-3activation (red), and plasma membrane permeabilization (pink) before and after treatment with 50 µg/mL of gallic acid for 1.5, 3, 4, 6, 7 and 9 hours (h). (Mean ± s.d.; *n* = 3; * *p* < 0.05).

It is generally believed that GA triggers cancer cells to die through apoptosis [20], which is a cell suicide process [21]. In the present study, we applied 50 µg/mL of GA to trigger cancer cell death, as *in vivo* studies demonstrated that administration of this dosage of GA is well-tolerated by animals, such as rats, without adverse physiological response detected [22,23]. This is also the concentration commonly used in the *in vitro* studies of GA to promote cancer cell death and other cellular responses [20,24,25]. Interestingly, we found non-typical apoptotic morphology in the GA-induced HeLa cells. Untreated HeLa cells were flat, spreading their cytoplasm on the substrate, and the nucleus was round (Figure 1A(*i*) and Figure S1A). In contrast, after being exposed to GA at 50 µg/mL for 12 h, the nuclei were condensed (Figure 1A(*ii*)), a hallmark of apoptosis [21,26,27]. However, the treated cells did not show two other key morphological hallmarks of apoptosis, namely plasma membrane blebbing and cell shrinkage (Figure S1B); instead, there was plasma membrane rupture (Figure 1A(*ii*)). This observation suggested that GA could induce other forms of cell death in addition to apoptosis in HeLa cells.

To confirm whether this observation in HeLa cells can be found in other cancer cells, we also examined GA-induced cell death in small cell lung cancer H446 and neuroblastoma SH-SY5Y cell lines by detecting multiple morphological and biochemical hallmarks of apoptosis. We found that in response to GA-induction at 50 µg/mL for 12 h, the HeLa, H446, and SH-SY5Y cells displayed the morphological hallmarks [26], including nuclear condensation and mitochondrial fragmentation, and also the biochemical hallmark [28], such as caspase-3 activation for apoptosis (Figure 1B). Interestingly, in line with what we had observed (Figure 1A(*ii*)), the GA-induced HeLa, H446, and SH-SY5Y cells also displayed plasma membrane rupture (Figure 1B), which is not the hallmark of apoptosis. This observation prompted us to investigate which form of cell death GA could trigger in cancer cells in addition to apoptosis.

### 2.2. Activation of Necroptotic Pathway by Gallic Acid

Apoptosis is also characterized by the maintenance of plasma membrane integrity in the apoptotic cells [26,27,29,30]. In contrast, necrosis is characterized by plasma membrane rupture and cell swelling [27,31]. Secondary necrosis refers to the plasma membrane rupture that occurs in dead apoptotic cells that have not been engulfed by neighboring cells or macrophages; such a loss of plasma membrane integrity is gradual and passive, taking hours or even longer [32,33].

To determine whether the plasma membrane rupture of GA-induced HeLa cells was attributed to secondary necrosis, we performed time-lapse live-cell microscopy to study the timeline of apoptotic events and plasma membrane rupture during GA-induction by using HeLa cells stably expressing cytochrome *c*-GFP (Figure 1C, and Supplementary Materials Video S1). These cells were stained with DNA binding blue dye Hoechst 33342 to label nuclear morphology, and then incubated in the culture medium with NucView 530 Caspase-3 red substrate to detect caspase activity, and also IncuCyte Cytotox reagent in deep red to detect plasma membrane rupture and permeabilization (Figure 1C,D and Supplementary Materials Video S1). Before apoptosis occurred, the cytochrome *c*-GFP was located in the mitochondria, which displayed a tubular network structure in the cells (Figure 1C(*i*),D) [34,35]. During early stages of GA-induction, the mitochondria fragmented (Figure 1C(*ii*),D), shortly followed by the cytochrome *c* release from mitochondria to cytosol (Figure 1C(*ii–iv)*,D), indicating the activation of a mitochondria-dependent apoptotic pathway [34,35,36,37]. The cytochrome *c* released cells then displayed nuclear condensation as visualized by the Hoechst 33342 blue nuclear stain (Figure 1C(*iv–vi*),D), and caspase activation as labeled by the NucView 530 Caspase-3 red substrate (Figure 1C(*vi’–viii’*),D). All four of them are the hallmarks of apoptosis [21,26,27]. Interestingly, plasma membrane rupture occurred shortly after caspase activation, indicated by the membrane rupture that was labeled by the membrane exclusion IncuCyte Cytotox reagent in deep red (Figure 1C(*vii’’*–*viii’’*),D). These results suggested that the plasma membrane rupture was mediated by an active cellular process other than the passive phenomenon of secondary necrosis.

While plasma membrane rupture can also occur in both necrosis and necroptosis, necrosis is defined as the uncontrolled cell death, and necroptosis (programmed necrosis) is a type of programmed cell death mediated by activation of the effector MLKL (mixed lineage kinase domain-like protein) for permeabilizing the plasma membrane [27,31,38,39].

**Figure 2:**
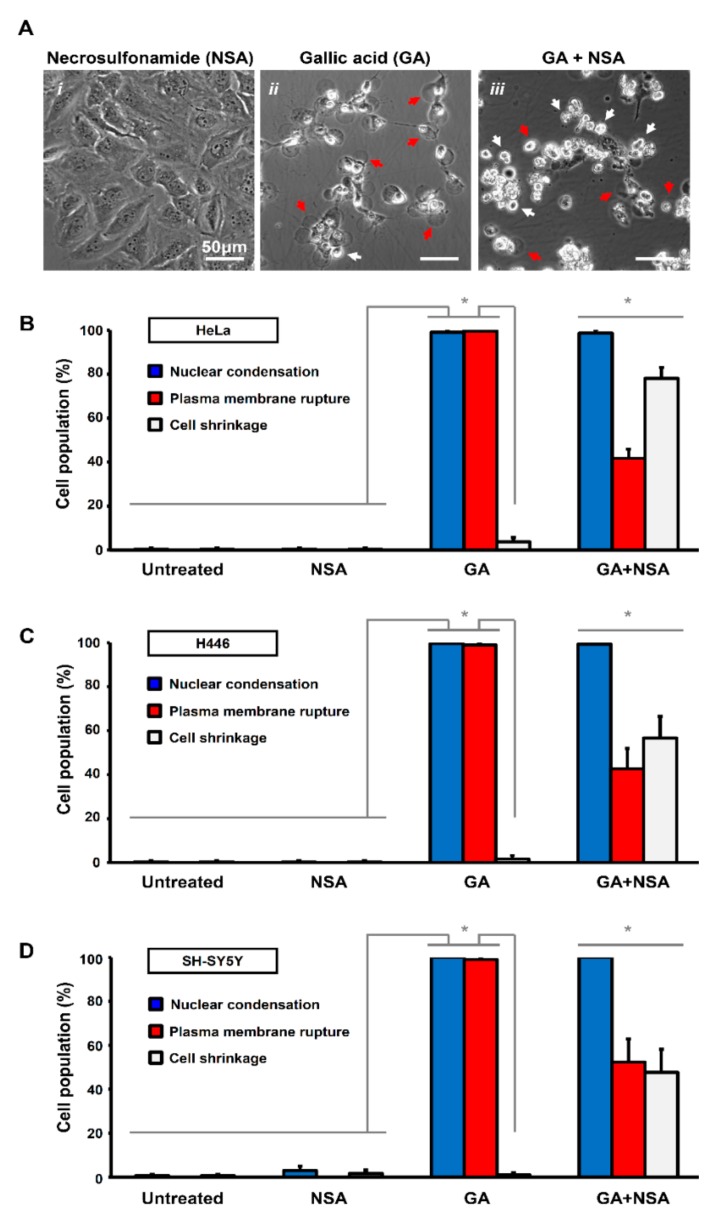
Necrosulfonamide suppresses plasma membrane rupture in gallic acid-induced cancer cells. (**A**) Representing confocal images of HeLa cells treated with necrosulfonamide (*i*. NSA, 5 µM), gallic acid (*ii*. GA, 50 µg/mL), and also co-treated with gallic acid and necrosulfonamide (*iii*. GA + NSA) for 12 h. Arrows indicate cells with plasma membrane rupture (red) and cell shrinkage (white). (**B**–**D**) Quantification of the cell death events in response to gallic acid induction with or without treatment with necrosulfonamide. The percentage in population of HeLa (**B**), H446 (**C**), and SY-SY5Y (**D**) cells displayed nuclear condensation (blue), plasma membrane rupture (red), and cell shrinkage (white) before or after gallic acid induction (50 µg/mL) with or without co-treatment with necrosulfonamide (NSA, 5 µM) for 12 h (Mean ± s.d.; *n* = 3; * p < 0.05).

To determine which form of cell death is executed in response to the GA-induction, we applied to HeLa cells the specific MLKL inhibitor, necrosulfonamide (NSA, 5 µM) [40], together with GA. As a control, HeLa cells exposed to NSA alone had normal cellular morphology, indistinguishable from the untreated control cells (Figure 2A(*i*)). Interestingly, we found that although NSA significantly suppressed plasma membrane rupture in the GA-induced cells, these cells displayed cell shrinkage as the hallmark of apoptosis (Figure 2A(*ii*–*iii*),B). These phenomena were also observed in the H446 and SH-SY5Y cell lines (Figure 2B–D). As NSA is an inhibitor of necroptosis [27,31] and suppresses GA-induced plasma membrane rupture, our results indicated that necroptosis occurred during GA-induction.

Interestingly, NSA did not suppress GA-induced cell death, as the cells co-treated with NSA and GA for 12 h still displayed the hallmarks of apoptosis, such as cell shrinkage and also nuclear condensation (Figure 2A(*iii*),B–D), thereby suggesting that both apoptosis and necroptosis occurred simultaneously during GA-induction. Noticeably, plasma membrane rupture was still observed at the prolonged treatment of GA for 36 h even with the presence of NSA (Figure S2), possibly due to second necrosis as a passive process to occur in dead cells [32,33]. Taken together, these findings indicated that GA could induce both apoptosis and necroptosis.

### 2.3. Activation of Ferroptotic Pathway by Gallic Acid

**Figure 3:**
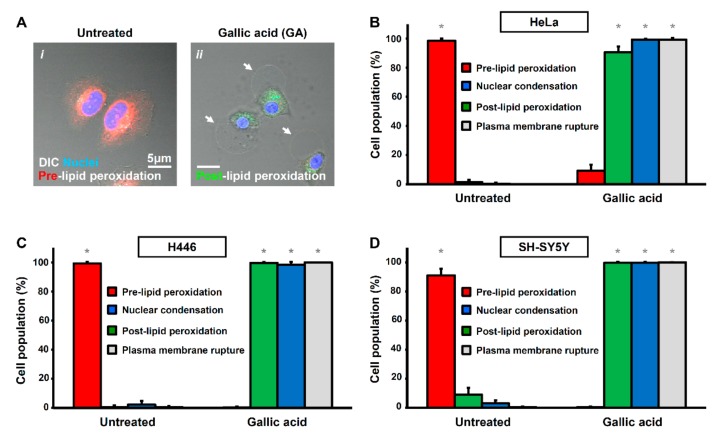
Gallic acid-induced cancer cells display hallmarks of apoptosis, ferroptosis, and necroptosis. (**A**) Representing confocal images of HeLa cells before (*i*) and after (*ii*) treatment with gallic acid (50 µg/mL) for 12 h. Cells were stained with Hoechst 33342 to visualize nucleus, and also lipid peroxidation sensor to detect pre-lipid peroxidation (red) and post-lipid peroxidation (green) for confocal microscopy. Cell morphology was observed by DIC microscopy. White arrows indicate the plasma membrane rupture. (**B**–**D**) Quantification of the cell death events after gallic acid induction. The percentage in population of HeLa (**B**), H446 (**C**), and SY-SY5Y (**D**) cells displayed pre-lipid peroxidation (red) and post-lipid peroxidation (green), nuclear condensation (blue) and plasma membrane rupture (grey) with or without treatment with gallic acid (50 µg/mL) for 12 h (Mean ± s.d.; *n* = 3; * *p* < 0.05).

**Figure 4:**
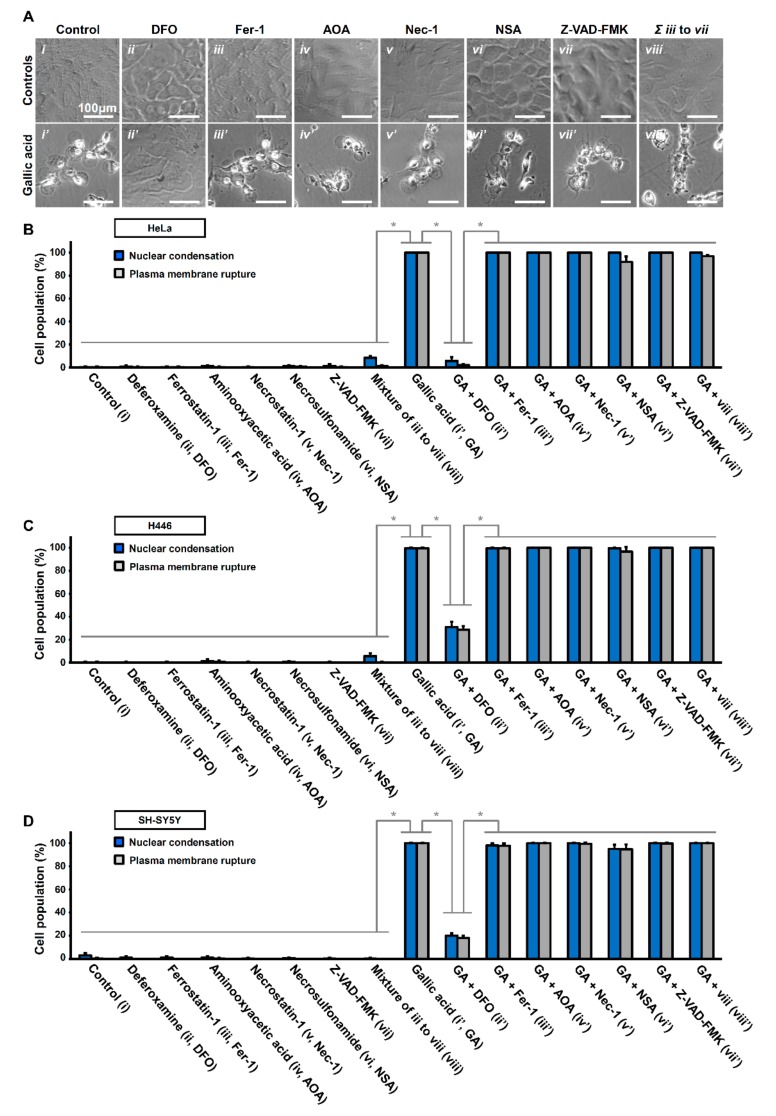
Iron chelator deferoxamine suppresses gallic acid-induced cell death. (**A**) Representing confocal images of HeLa cells treated with (*i*) medium alone (Control), (*ii*) deferoxamine (DFO, 200 µM), (*iii*) ferrostatin-1 (Fer-1, 2 µM), (*iv*) aminooxyacetic acid (AOA, 2 mM), *v*) necrostatin-1 (Nec-1, 40 µM), (*vi*) necrosulfonamide (NSA, 5 µM), (*vii*) Z-VAD-FMK (50 µM), and (*viii*) co-treated with Fer1, AOA, Nec-1, NSA, and Z-VAD-FMK, with (lower panels, (*i’*–*ix’*)) or without (upper panels,(*i*–*ix*)) co-treatment of gallic acid (50 µg/mL) for 36 h. Cell morphology was observed by DIC microscopy. (**B**–**D**) Quantification of the cell death events in HeLa cells after gallic acid induction (50 µg/mL) with or without co-treatment with inhibitors with the conditions listed at the panel A for 36 h. The percentage in the population of HeLa cells displayed nuclear condensation (blue) and plasma membrane rupture (grey) as mentioned above in HeLa (**B**), H446 (**C**), and SY-SY5Y (**D**) cells. (Mean ± s.d.; *n* = 3; * *p* < 0.001).

We further identified ferroptosis to occur in GA-induced cell death. Ferroptosis is characterized by lipid peroxidation as a form of oxidative stress [41,42,43]. Lipid peroxidation was not detected in healthy HeLa cells without exposure to GA (Figure 3A(*i*)). However, HeLa cells did show lipid peroxidation in response to GA induction for 12 h (Figure 3A(*ii*)). Interestingly, these cells also displayed the hallmarks of apoptosis and necroptosis, characterized by nuclear condensation and plasma membrane rupture, respectively (Figure 3A(*ii*)). Our quantification study also confirmed these observations in the major population of HeLa, H446, and SH-SY5Y cell lines (Figure 3B–D). Ferroptosis is also defined as an iron-dependent form of cell death [27,41], which can be suppressed by the iron chelator deferoxamine (DFO, 200 µM) [41,43]. Therefore, to determine if GA activates ferroptotic cell death pathway, we applied DFO together with GA. We found that DFO could suppress GA-induced cell death in HeLa, H446, and SH-SY5Y cells (Figure 4A(*i*–*ii*),(*i’*–*ii’*),B–D), as the cells co-treated with GA and DFO retained their normal cellular morphology (Figure 4A(*i–ii*),(*i’–ii’*)). Taken together, our results indicated that GA could trigger apoptosis, ferroptosis, and necroptosis in the same cells simultaneously.

### 2.4. Other Unidentified Cell Death Mechanisms Triggered by Gallic Acid

We were curious if the GA-induced cell death was solely mediated by apoptosis, ferroptosis, and necroptosis, or if other cell death mechanisms were involved. If the former, GA-induced cell death should be suppressed by the combined treatment of inhibitors specifically targeting these three forms of cell death mechanisms. To answer this question, we, therefore, co-treated HeLa cells with the potent apoptosis inhibitor pan-caspase inhibitor Z-VAD-FMK (50 µM), the ferroptosis inhibitors ferrostatin-1 (Fer-1, 2 µM) and aminooxyacetic acid (AOA, 2 mM), and the necroptosis inhibitors necrostatin-1 (Nec-1, 40 µM) and MLKL inhibitor necrosulfonamide (NSA, 5 µM).

We found that HeLa cells treated with these inhibitors individually or co-treated all together did not show any signs of cell death and were comparable to the untreated control cells (Figure 4A(*iii–viii*)). Co-treatment with one of these inhibitors together with GA did not suppress GA-induced cell death (Figure 4A(*iii–vii*)), also as expected, because targeting one of the three identified GA-induced cell death pathways is insufficient to suppress the rest of the two cell death processes for cell demise. Furthermore, simultaneous treatment with all three kinds of inhibitors was still unable to inhibit GA-induced cell death (Figure 4A(*viii’*)). Our quantification study confirmed these observations not only in HeLa cells but also in the H446 and SH-SY5Y cell lines (Figure 4B–D). This indicates that GA triggers one or more cell death mechanisms other than apoptosis, necroptosis, and ferroptosis, that remain(s) to be identified.

Noticeably, DFO could suppress GA-induced cell death, as mentioned above (Figure 4A(*ii’*)), in all three cell lines (Figure 4B–D). These results reveal that GA triggered an iron-dependent cell death mechanism, which is upstream of the apoptotic, ferroptotic, and necroptotic cell death pathways.

### 2.5. Synergistic Effect of Necrosulfonamide and Gallic Acid in Cell Death Induction

**Figure 5:**
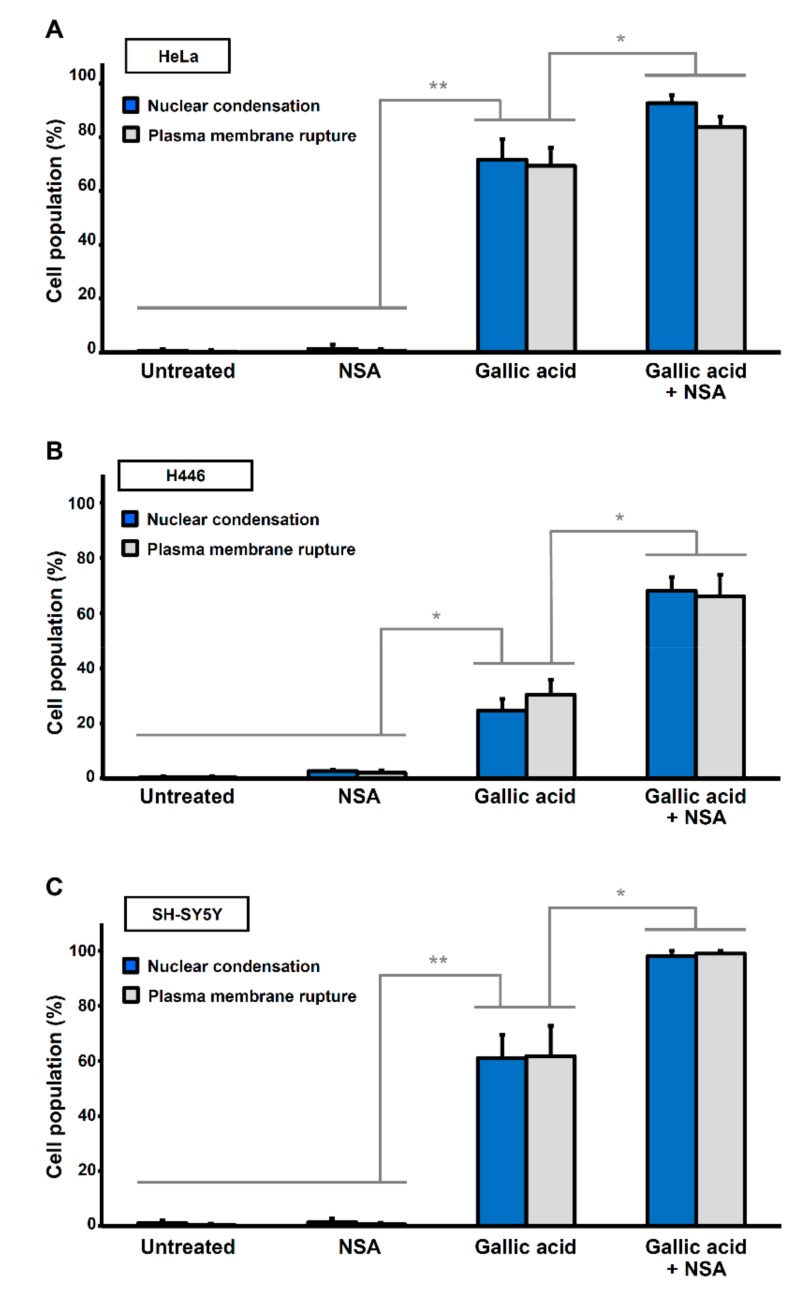
Necrosulfonamide promotes gallic acid-induced cell death. Quantification of the cell death events in (**A**) HeLa, (**B**) H446, (**C**) SH-SY5Y cells in response to gallic acid induction (30 µg/mL) for 36 h with or without co-treatment with necrosulfonamide (NSA, 2 µM). The percentage in the population displayed nuclear condensation (blue) and plasma membrane rupture (grey). (Mean ± s.d.; *n* = 3; * *p* < 0.05; ** *p* < 0.02).

Although necrosulfonamide (NSA) could not suppress GA-induced cell death, we discovered an unexpected synergistic effect, by demonstrating that NSA enhanced the sensitivity of cancer cells to GA-induced cell death. At a lower concentration of GA (30 µg/mL) that killed only 70% of HeLa cells, application of NSA (5 µM) in a condition that would not trigger cell death alone, resulted in a cell death above 90% when both GA and NSA were combined to treat the cells (Figure 5A). This synergistic effect was also observed in the H446 and SH-SY5Y cells (Figure 5B,C). Taken together, these findings revealed a possible strategy to promote the anti-cancer effect of GA at a lower dosage in the presence of NSA.

## 3. Discussion

**Figure 6:**
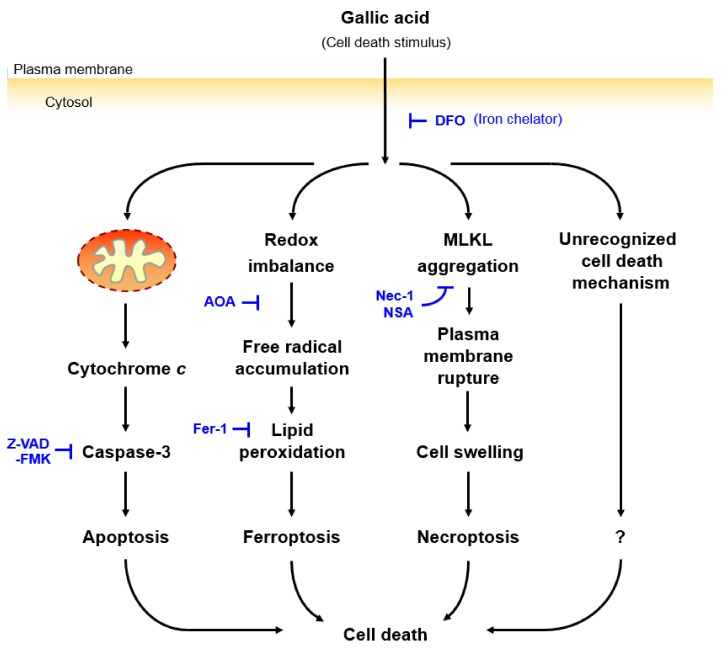
Proposed mechanisms of gallic acid-induced cell death. Gallic acid triggers cell death with activation of apoptotic, ferroptotic, and necroptotic pathways. These forms of cell death can be suppressed by the iron chelator DFO, indicating that such cell death is iron-dependent. However, the gallic acid induced cell death cannot be suppressed by the inhibitors targeting three specific cell death pathways, such as the apoptosis inhibitor Z-VAD-FMK, the ferroptosis inhibitors AOA and Fer-1, and the necroptosis inhibitors Nec-1 and NSA, even with combined treatment. This result implies that there is an unrecognized downstream pathway for executing cell death.

It is generally believed that GA promotes cell death through apoptosis [20]. However, we presented the first evidence that GA activated not only the apoptotic cell death pathway; it also activated ferroptotic and necroptotic cell death pathways in the same cells (Figure 6). We have determined that this GA-induced cell death was iron-dependent, as the application of the iron chelator DFO alone could suppress the GA-induced cell death. Interestingly, inhibitors targeting the downstream regulators of apoptosis, ferroptosis, and necroptosis, alone or in combination, did not abolish GA-induced cell death, suggesting that other cell death mechanisms could be involved, and these remained to be identified. We also demonstrated that the necroptosis inhibitor NSA could promote GA-induced cell death via a synergistic effect.

Our study provides new insights into the development of new anti-cancer treatments with a lower side effect. Adverse side effects are the major drawback of many current anti-cancer treatments [6,7,8]. Studies in human cancer xerograph mouse models have demonstrated the anti-cancer effect of GA with low side effects [20]. Here, observation of the anti-cell death effect of DFO, and pro-cell death effect of NSA, in GA-induction revealed a new strategy that could enhance the anti-cancer treatment of GA by further lowering its potential side effects. For example: (i) DFO could be locally delivered to the normal tissues surrounding the tumor side, protecting these normal tissues from cell death induction during the GA treatment, thereby reducing the side effects, and (ii) by using controlled drug delivery [44], NSA could be delivered to the tumor side to enhance the anti-cancer effect with a lower dosage of GA.

Our study also leads to at least three key unanswered questions: Which form(s) of unidentified cell death mechanism could be triggered by GA? How can NSA promote GA-induced cell death? Why are cancer cells more sensitive to GA than healthy cells? Although the iron chelator DFO could suppress GA-induced cell death, none of the inhibitors targeting the downstream effectors of apoptosis, ferroptosis, or necroptosis could do so. This finding indicates that the unidentified cell death mechanism(s) being activated, was (or were) mediated by an iron-dependent pathway. Furthermore, there could be the cross-talk between different cell death pathways [27,45], in which suppression of the necroptotic pathway by NSA could strengthen other cell death mechanisms to which cancer cells are highly sensitive. It is also possible that GA could activate a currently unidentified cell death mechanism that is present in cancer cells but not in healthy cells, thereby leading to the difference in their sensitivity toward GA.

There are challenges in testing these exciting hypotheses, including the paucity of cell death markers and specific targeting agents for manipulation. While over 20 forms of programmed cell death have been identified [27], they are not equally well characterized. The most well-characterized cell death mechanisms are apoptosis, ferroptosis, and necroptosis, for which there are numerous studies on their hallmarks of cell death and the underlying molecular mechanisms, and for which specific inhibitors targeting their key regulators have been identified [27].

Our present study also provides new cell models with which to study the crosstalk between cell death pathways, including apoptosis, ferroptosis, necroptosis, and other mechanisms that have not yet been characterized here for GA-induced cell death. Future research for discovering and characterizing new forms of cell death will continue to advance our knowledge of the molecular mechanisms and key regulators that mediate cell death induced by GA, the identification of which should provide new tools and approaches for treating cancer.

## 4. Materials and Methods

### 4.1. Chemicals

Aminooxyacetic acid (C13408-1G), deferoxamine (D9533-1G), dimethyl sulfoxide (D2438), ferrostatin-1 (SML0583-5MG), gallic acid (G7384-100G), necrostatin-1 (N9037-10MG), and necrosulfonamide (480073-25MG) were from Sigma-Aldrich (Burlington, MA, USA). Z-VAD-FMK (ALX-260-020-M005) was purchased from Enzo Life Sciences (Farmingdale, NY, USA).

### 4.2. Cell Culture

Three human cell cancer lines were used in this study. The human cervical cancer HeLa, and neuroblastoma SH-SY5Y cell lines were cultured in DMEM/F-12 medium, while the small cell lung cancer H446 cell line was cultured in RPMI medium. Both culture media were supplemented with 10% fetal bovine serum (FBS), 100 U/mL penicillin, and 100 μg/mL streptomycin (Thermo Fisher Scientific, Carlsbad, CA, USA). Cells were maintained at 37 °C under an atmosphere of 5% CO_2_. Cells were seeded on 35 mm glass-bottom culture dishes (MatTek Corporation, Ashland, MA, USA) for 24 h before the experiment began. Cells were seeded to culture dishes for 24 h to achieve 50–60% confluence before subjected to experiment.

### 4.3. Cell Death Induction

For induction of cell death, GA was first diluted in dimethyl sulfoxide (DMSO) to make a stock solution with 20 mg/mL, and then further diluted into the cell culture medium to achieve the working concentration as stated in the figure legends of the corresponding experiments. The inhibitors of apoptosis (Z-VAD-FMK), ferroptosis (aminooxyacetic acid, deferoxamine, and ferrostatin-1), and necroptosis (necrostatin-1, and necrosulfonamide) were first diluted in DMSO or water, as recommended by manufacturers, and then mixed with the cell culture medium to achieve the working concentration as stated in the figure legends before being applied to the cells.

### 4.4. Live Cell Staining

Cells were seeded on 35 mm glass-bottom dishes (MatTek Corporation, Ashland, MA, USA) for 24 h before subjected to live cell staining. Nuclei were stained with 10 μg/mL of Hoechst 33342 blue nuclear dye in the culture medium, for 20 min at 37 °C with 5% CO_2_. The stained cells were then washed twice with fresh medium and incubated for a further 10 min before the microscopy observations.

For detecting the caspase-3 activity using the NucView 530 Caspase-3 substrate (#10406) from Biotium (Fremont, CA, USA), the substrate was diluted with cell culture medium 1:2000, and then applied to the cells and incubated at 37 °C under an atmosphere of 5% CO_2_ for 20 min before subjected to microscopy.

For detecting plasma membrane permeabilization with IncuCyte Cytotox Red reagent (#4632) from IncuCyte (Göttingen, Germany), the reagent was applied to the culture medium to achieve a 1:1000 dilution and incubated for 20 min before microscopy.

For detecting lipid peroxidation with Image-iT™ Lipid Peroxidation Kit (C10445) from Thermo Fisher Scientific(Carlsbad, CA, USA), the reagent was applied to the culture medium to achieve a 1:1000 dilution, incubated for 30 min, and then washed twice with fresh culture medium before microscopy.

### 4.5. Confocal Microscopy

Time-lapse confocal microscopy of live cells was performed by placing a 35 mm culture dish on the stage top incubator of an inverted confocal microscope LSM780 (Carl Zeiss, Jena, Germany), equipped with lasers with excitation wavelength 405 nm (DAPI), 488 nm (FITC), 561 nm (Cy3), and 633 nm (Cy5). Cell images were obtained using 10× Plan-Neofluar objective with a numerical aperture (N.A.) 0.30, or a 40× Plan-Apochromat objective with N.A. 1.4, by imaging cells from the bottom of the dish through a glass coverslip. Cells were incubated at 37 °C with 5% CO_2_ throughout the live-cell imaging process. The images were analyzed and edited using Zen and AxioVision software (Carl Zeiss, Jena, Germany).

### 4.6. Statistical Analysis

Statistical comparison was performed using two-tailed Student’s *t*-test. At least three biological replicates for each experiment were performed, and 100 cells were counted for each replicate. The results are shown as the mean ± standard deviation (s.d.). Differences are considered to be significant when the *p*-value is <0.05.

## Supplementary Materials

The following are available online at https://www.mdpi.com/2072-6651/11/9/492/s1, Figure S1: Morphology of apoptotic HeLa cells, Figure S2: Necrosulfonamide cannot suppress plasma membrane rupture at the gallic acid-induced prolonged treatment, Video S1: Gallic acid-induced HeLa cells.

## Abbreviations

Aminooxyacetic acid	AOA
Cytochrome *c*	Cyto*C*
Deferoxamine	DFO
Differential Interference Contrast	DIC
Dimethyl Sulfoxide	DMSO
Ferrostatin-1	Fer-1
Gallic acid	GA
Low Molecular Weight	LMW
Necrostatin-1	Nec-1
Necrosulfonamide	NSA
