# Supplementary material for: Gallic Acid Triggers Iron-Dependent Cell Death with Apoptotic, Ferroptotic, and Necroptotic Features
Source: Toxins (Basel). 2019 Aug 26;11(9):492. doi: 10.3390/toxins11090492 (PMC6783835; doi:10.3390/toxins11090492)
Supplement: Supplementary file 1 [file toxins-11-00492-s001.zip › toxins-571464-Suppl.docx]

Supplementary Materials: Gallic Acid Triggers Iron-Dependent Cell Death with Apoptotic, Ferroptotic, and Necroptotic Features

Ho Man Tang and Peter Chi Keung Cheung

**Figure S1.** Morphology of apoptotic HeLa cells. Live-cell confocal microscopy of human cervical cancer HeLa cells before cell death induction (**A.** Untreated), and treated with cell death inducer 3.9% ethanol for 3 h (**B.** Treated). Merged images of MitoTracker-stained mitochondria (red) and the Hoechst-stained nucleus (blue) were visualized by confocal microscopy, and cell morphology by DIC microscopy. Scale bar, 10 μm.

**Figure S2.** Necrosulfonamide cannot suppress plasma membrane rupture at the prolonged treatment of gallic acid induction Representing confocal images of HeLa cells co-treated with necrosulfonamide (5 µM) and gallic acid (50 µg/mL) for 36 h. Arrows indicate cells with plasma membrane rupture (red) and cell shrinkage (white).

**Video S1.** Time-lapse live-cell confocal microscopy on cytochrome *c*-GFP expressing HeLa cells treated with 50 µg/mL of gallic acid. Merged images of differential interference contrast (DIC) microscopy, cytochrome *c*-GFP (Cyto*C*, green), nucleus (blue), caspase activity (red), and plasma membrane-permeable dye (pink). Cells were stained with blue nuclear dye Hoechst 33342, before treatment with gallic acid together with NucView 530 Caspase-3 substrate (red) and plasma membrane-permeable dye IncuCyte Cytotox red reagent (pink). This video is the supplementary material of Figure 1C. The video is composed of 228 films, played as 10 films per second, with 3 min as original time per interval between each of the acquired films.
